# Catheter ablation vs. anti-arrhythmic drug therapy for ventricular tachycardia in ischaemic heart disease—Authors’ reply

**DOI:** 10.1093/europace/euag141

**Published:** 2026-06-15

**Authors:** Francesco Santoro, Laura Perrotta, Giacomo Mugnai

**Affiliations:** Cardiology Unit, Department of Medical and Surgical Sciences, University of Foggia, Italy; Arrhythmia Unit, Department of Cardiology, Careggi University Hospital, Florence, Italy; Division of Cardiology, Cardio-Thoracic Department, University Hospital of Verona, Verona, Italy

We thank Griné et al. for their interest in our meta-analysis and for their thoughtful comments regarding endpoint definition and trial selection.^1^

Regarding the primary endpoint, we acknowledge that the reporting of appropriate ICD therapies was not fully homogeneous across the included randomized trials. As specified in the Methods section, the primary endpoint was defined according to the original trial definitions and available published data. In some studies, detailed separation between anti-tachycardia pacing and ICD shocks was not consistently reported, reflecting an intrinsic limitation of the currently available randomized evidence. Nevertheless, all included studies evaluated clinically relevant ICD-treated ventricular arrhythmias, supporting the overall consistency of the pooled analysis.

Concerning trial selection, we agree that SMASH-VT^2^ and VANISH^3^ differed in design and therapeutic strategy. However, both trials addressed clinically relevant ablation-based vs. anti-arrhythmic drug-based management approaches in patients with ischaemic heart disease and ventricular arrhythmias. For this reason, we considered their inclusion justified and representative of contemporary real-world management strategies. Importantly, despite differences in study design, the direction of treatment effect remained consistent across trials.

As highlighted in the Discussion, moderate heterogeneity was present and mainly related to differences in trial design, timing of ablation, and background anti-arrhythmic therapy. We agree that future patient-level analyses and additional randomized studies may further refine the comparative role of catheter ablation and anti-arrhythmic drugs in this complex population.

We thank the authors again for their valuable contribution and for the opportunity to further discuss this important topic.
